# Pathology

**Published:** 1826-04

**Authors:** 


### PATHOLOGY.

Actio læsa suum designat singula morbum;
Præterea morbum partis mutatio signat.

6. *M. Louis on Phthisis.** The reporters observe, the subject of pulmonary phthisis has been so ably and so minutely handled by M. Bayle, that whoever pretends to follow the same path must expect to have some unfavourable prepossessions to overcome. Arrived at that age when physicians generally merge all ardour for science, in the ac-

* Rapport fait à l'Academie Royale de Médécine, sur un Manuscrit intitulé, "Recherches Anatomico-Pathologiques sur la Phthisie." Par M. Louis.

quisition of wealth, M. Louis gave up private practice, and devoted himself to a laborious investigation, which is by no means yet exhausted.

From the month of October, 1821, up to the date of publication, he has dedicated the whole of his time to the histories, treatment, cures, deaths, and dissections of the patients admitted into the wards of La Charité, amounting in that period to the number of 1960, of whom 358 died. Of these last, there were 127 deaths by phthisis, and 40 by other diseases, the patients still presenting tubercles in the lungs. This morbid phenomenon then existed in nearly one half of all who died in the hospital—a fearful proportion of tubercular disposition—constituting the principal, if not the exclusive cause of death in more than one case out of three! Now, as it is not probable that a greater proportion of tubercular diseases should enter the Charité than any other hospital, it is reasonable to conclude that this proportion would hold good throughout all the public establishments, of general reception, and, possibly, through private life—and death. Should this be the case, the tubercular disposition is more predominant in France than in this country, according to our present calculations:—For we believe it has never been computed that more than one in four or five die of tubercular phthisis in England.

The first part of M. Louis's work is dedicated to the *anatomical lesions*; and, as these had been so well described by Bayle, Laennec, and other writers, our author is obliged to be very concise. Yet even this part is not destitute of original observations. Thus, for example, he has proved that not only do tubercles affect especially the *superior portions* of the lungs, but that, when they are found in the different lobes, those situated in parts above-mentioned, are always more numerous, larger, and sooner suppurated than the others. He has often found the upper lobe entirely disorganized by tubercles, while the inferior was either free, or nearly so, from tuberculation.

The air-passages have offered to our author some morbid phenomena, imperfectly described by Bayle. In one hundred and two cases, M. Louis has found 18 instances of ulceration of the epiglottis—23 of laryngeal ulceration, and 31 of ulceration of the trachea. In several instances he found the tracheal ulceration occupying the whole of the muscular portion of this conduit; and, in one case, several of the cartilaginous rings completely destroyed. In respect to the mucous membrane of the lungs, our author has found it but seldom affected, even in the neighbourhood of *crude* tubercles; while, in the vicinity of excavations, especially if large, or of long standing, this membrane was almost always thickened and red. He thinks the phlogosis of the mucous membrane is the effect, and not the cause, of the tubercular excavations.

In about a tenth of the whole number, it appeared to our author that acute inflammation took place in the parenchymatous substance of the lungs during the last days of the patient's existence.

The pleural adhesions, so common in phthisical patients, have attracted much of our author's attention. He only found a single case where both lungs were free from these adhesions. There was generally a relative proportion between the degree of the adhesions and the size and number of the excavations.

In nearly a third of those who died of tubercular phthisis, our author found tubercles or tuberculous matter in the *small intestines*. In one case in nine, the *large intestines* offered similar phenomena. In a *fourth part* of the number the mesenteric glands were tuberculous—in a tenth, the cervical glands—in a twelfth, the lumbar lymphatic glands—in a thirteenth, the prostate was tuberculous—in a fourteenth, the spleen—in a twentieth, the ovaries—in a fortieth, the kidneys—in one instance only was the brain, spinal marrow, or ureters affected with tuberculation.

This investigation, conducted with such scrupulous exactness, has led our author to the following important result, viz. that in no one instance, where tuberculation obtained in any one organ of the body, did the same morbid phenomenon fail to appear in the lungs. Even in every case, where tuberculous concretions formed in the serous membranes, as the result of chronic inflammation, there was *pulmonary* tuberculation in the same subject. The lungs then exhibit a most remarkable disposition to this deplorable malady.

The researches of M. Louis have corrected an error into which several pathologists have fallen respecting enlargements of the right chambers of the heart. Many physicians have considered pulmonary tuberculation as a fertile source of diseases of the heart in general, and particularly of enlargements of the right ventricle. It was supposed that tubercles caused obstruction to the circulation, and that this last led to dilatation of those chambers of the heart connected with the pulmonary circulation. This supposition is negatived by M. Louis, who found in the great majority of those who fell under phthisis, the heart and aorta *diminished* in size, in proportion to the shrinking of the other viscera, the usual consequences of the pulmonic emaciation.

In ninety-six cases, (phthisical) where the stomach was carefully examined, there were found nine instances wherein the volume of this organ was doubled or even tripled. Only two examples of this phenomenon were observed among all the patients who died of other diseases. But other lesions of the stomach were very numerous among the phthisical dissections. Seventy-eight out of the ninety six, presented strong marks of disease, such as softenings of the coats—extenuation—redness with thickening, &c. &c. In two cases there were ulcerations, without any other change of structure in this organ. It is, therefore, evident that affections of the stomach are very frequent in the latter stages of phthisis pulmonalis. The same may be said, and still more strongly, of ulceration of the small intestines. Five in six of those who died of phthisis presented this lesion. It is curious that wherever this ulceration of the mucous membrane existed, a corresponding thickening, either of the cellular or muscular membrane obtained, as if Nature took this precaution to guard against erosion of the canal. The cœcum, colon, and rectum did not quite so frequently present the lesion in question. This affection of the mucous membrane accounts for the general finale to consumptive cases—colliquative diarrhœa.

In two-thirds of the dissections M. Louis found that fatty condition of the liver ("l'etat graisseux du foie") described by M. Bayle. This state seems almost peculiar to tubercular phthisis, for, in the examination of 220 bodies, who had died of other diseases, only two instances of this kind were observed.

Such is an outline of the first part of M. Louis's work. The second part is on the prominent symptoms of tubercular phthisis, and presents some curious and important results.

In two-thirds of the cases of phthisis the disease was preceded by hæmoptysis. In one-fifth of the number this accident happened before there was any cough or expectoration. In the whole 1960 individuals there were none who had spontaneous pulmonary hæmorrhage, except the phthisical portion. The exceptions were a very small number of females who had discharges of blood from the chest in suppressions of the catamenia. From these premises the author concludes that pulmonary hæmorrhage, to any extent, is, in itself, a strong presumption that there are tubercles in the lungs.*

The pains in the chest which are complained of, may be attributed either to the pulmonary tubercles, or to the adhesions; but, as these two phenomena are almost always found together, it is difficult to say which of them is the cause of the pains. It is infinitely more probable, however, that the pains are owing to the pleural adhesions than to the tuberculous growths in the pulmonary structure.

*Cold chills*, or rigors were observed in five-sixths of the cases—night-sweats in nine-tenths. The alternations of night-sweats and diarrhœa were not found in near so regular a succession as both ancient and modern authors have laid down.

*Diarrhœa* shewed itself, for a longer or shorter period, in almost all the patients who sunk under phthisis. In proportion to the length of time which it continued before death, was the degree of ulceration in the mucous membrane of the intestines.

Those patients in whom ulceration of the epiglottis was found, had experienced a fixed pain at the upper part of, or rather above, the thyroid cartilage—and difficulty of deglutition, sometimes so great as to cause liquids to be returned through the nostrils.

Local pain, more or less acute, and complete aphonia, during one or more months, were the only *characteristic* symptoms of *laryngeal* ulceration. As to ulcerations of the trachea, they were not accompanied by any *particular* symptoms on which our author could ground a specific diagnosis.

The lesions of the stomach were denoted by apyrexia, epigastric uneasiness, nausea, and even vomiting. Gastric affection being so very common a phenomenon in phthisis, and this last being, unfortunately,

---

* The Commission, who report on this work, consider this conclusion of our Author's as greatly invalidated by the experience of the profession. We confess we are gloomy enough to believe that M. Louis is very near the truth, in the above deduction.—*Rev.*

so very prevalent in all northern climates, it was very desirable to ascertain what connexion existed between the state of the tongue and the state of the stomach, in the latter stages of phthisis—this connexion being so much insisted on by modern writers, and especially by the numerous adherents of the school of Broussais. Voici donc.

Out of 96 cases of the most accurate dissection of the stomach in phthisical patients, this viscus was found without the slightest appreciable lesion in nineteen instances. In the other 79 cases, the stomach presented various lesions. Of the 19 cases abovementioned, nine presented, while living, more or less redness of the tongue. In one instance, this redness was in the highest degree, and had continued so during the whole of his sojourn in the hospital, which was 32 days before his death—yet, on dissection, the stomach was found perfectly natural in every respect. Of the 79 cases where there were various lesions of the stomach, 35 only presented redness of the tongue during life, and in five of these, the redness was extremely trifling, and only temporary. "It hence results that the redness of tongue was found in nearly an equal proportion among those who had, and those who had not, an affection of the mucous membrane of the stomach."

Every one knows how easy the diagnosis of phthisis pulmonalis is, at an advanced period of the disease—and how difficult is the said diagnosis in the early periods of the same! M. Louis has directed his attention much to this subject, and the following are his observations:—

"A dry cough, of considerable duration; a shortness of breath easily induced by exertion or speaking; pains more or less acute in the back or sides; a notable diminution of embonpoint and muscular strength; —these are symptoms which ought to lead us to suspect the existence of tubercles in the lungs. If they succeed one or two attacks of hæmoptysis, we may be almost certain that phthisis is at hand. Auscultation and percussion should then be had recourse to, in order to solve our doubts. If the sound of the chest is dull under one of the clavicles, for a small extent of surface—if the respiratory murmur is there feeble, and accompanied by some degree of wheezing, (râle) these phenomena being absent in other parts of the chest, then we have strong confirmations of our fears."

When the disease is advanced to that period when the tubercles become broken down, and excavations formed, then the diagnosis is sufficiently easy. Pectoriloquism furnishes a certain criterion, with the exception of those comparatively rare cases where bronchial dilation exhibits the phenomenon of pectoriloquism. The other symptoms will then generally decide the question.

Here we close this short, but we hope interesting coup d'œil of M. Louis's work, presuming that we have not misspent our own time nor that of our readers on the occasion.

---

7. *Angina Pectoris.* There can be little doubt that many cases are supposed to be angina pectoris which are very different from this dreadful disease. Thus, Dr. Lind, of Copenhagen, has related a case (Bibliothek for Laeger, ann. 1825) which he calls by this name, and which he cured by antimonial ointment to the chest, and a course of the tinct. guaiaci ammon. The patient had, for several years, especially in autumn and winter, suffered from severe oppression about the chest, and pains in the arms, for which bleeding, leeching, and various anti-spasmodic medicines had been tried in vain. Dr. L. used the means above-mentioned, and the patient recovered. He considered the affection a rheumatism of the cardiac plexus. Without attaching much importance to the case related by Dr. Lind, we are inclined to think that a neuralgic affection of the cardiac plexus may have something to do with the pathology of this dreadful disease, at least, in some cases. A recent example of angina pectoris, in one of its most terrible forms, lately occurred at Hampstead, and was seen by Dr. Latham, Dr. Maton, Dr. Bree, Mr. Rodd of Hampstead, and some other medical gentlemen, as well as Dr. Johnson, under whose care the patient was during the last 18 months of his life.

Mr. Wright was about 58 years of age, and with the exception of a nephritic complaint, had enjoyed general good health. About 18 months ago, while walking up to Hampstead, he felt an indescribable sense of stricture and pain across the chest, which caused him to stop for several minutes, when he recovered. These accessions became gradually more severe in degree, and returned at shorter intervals. They, also, soon became accompanied by pain darting sometimes from the region of the heart down along the left arm to the wrist, or even to the fingers. Dr. Johnson had the patient under his care from this time till death took place. The chest, examined carefully by percussion and by the stethoscope, appeared to contain no organic lesion. The heart beat regularly, though somewhat weakly, over the ordinary space. The disease was readily recognized to be angina pectoris, probably depending on a flabby and debilitated state of the muscular structure of the heart; which opinion was delivered to the other medical gentlemen who visited the patient in succession. For the last six months the paroxysms became dreadful in violence, duration, and frequency. They generally came on in the evening, and lasted, with more or less force, till five or six o'clock in the morning, during which time, the unfortunate patient was obliged to keep in the perpendicular posture, crying out with the pain in his arm and chest, and employing one or two people constantly rubbing his arm, breast, and back, in order to mitigate the pain. Opium, brandy, ammonia, ether, and the strongest antispasmodics and stimulants gave only very temporary, or oftener no relief. The longest interruption of the spasms (nearly a month) took place after the application of a large tartar-emetic plaster to the chest, which produced an extensive ulceration that required three weeks to heal. In this period he went to Ramsgate, where the spasms returned, and he came back to Hampstead worse than ever. It may be worthy of remark that, in this case, the pain occasionally arose in the arm, and darted thence into the chest, occasioning the most indescribable suffering there. At other times, the pain commenced about the heart, and flew to the arm, where friction, even with the handles of brushes or rough pieces of wood, was necessary for hours together! The urine now became scanty—the legs swelled—the patient was worn out with nights of agony and days of stupor and languor—and, in one of the paroxysms, death happily came, as a friend that had long been invoked by one of the greatest sufferers which the writer of this article ever saw.

On dissection, the lungs were observed to be perfectly sound. No water of any consequence, either in the bags of the pleura or the cavity of the pericardium. The heart was of the usual size, but much covered with fat. Its muscular structure was pale, flabby, and so lacerable as to be easily mashed between the fingers, like wetted paper or putrid meat. There was no disease of the valves, nor of any of the large vessels issuing from the heart. The left coronary artery was taking on an indurated or cartilaginous condition. The parietes of the ventricles were rather extenuated, as well as softened in structure. Mr. Rodd and two other medical gentlemen assisted Dr. Johnson in examining the body. There was no other disease, except an obliteration of the pelvis of the left kidney.

The above was an exquisitely marked case of the angina pectoris of authors, although only a few of the particulars are here stated, in consequence of the well-known characters of the disease. Mr. Wright was seen by several of the most eminent medical men in this metropolis, who all concurred that there was disease of the heart, though there were several opinions as to the precise pathological condition of this organ.

We have so often met with this softened structure, or flabby state of the heart, accompanied with those symptoms which characterize angina pectoris, that we consider this pathological condition as a very common cause of the disease. Whether or not there be any appreciable lesion of the nerves supplying the heart, in these cases, our dissections (being principally carried on in private practice) do not enable us to judge. It is incumbent on those in hospital practice, who have the leisure and opportunities for such minute researches, to investigate this point. Certainly the dreadful sufferings consequent on this disease, would lead one to suspect an affection of the nerves of the organ—but this is yet an unbeaten path of pathology.

---

8. *Gangrene of the Lungs.** Before the publication of Laennec on Auscultation, there were only vague notions of this comparatively rare disease. That estimable pathologist observes, that *general* gangrene of the lungs must seldom occur, as he has only seen two cases of it in 18 years. He describes the lungs, in this state, as being very easily torn—and of various shades of colour, from grey to a black, with some spots so sof-

* Sur la Gangrène des Poumons. Par M. Bouillaud. Revue Med. December, 1824.

tened as to appear putrid. When the lungs are cut into, a sanious, greenish, and most fetid fluid oozes out. This condition may occupy the greater part, or even the whole of a lobe, and is not circumscribed.

The partial gangrene of the lungs, according to Laennec, differs from the foregoing in being circumscribed in extent, and occupying but a very small space. The symptoms of this disease will be easily gathered from the cases which are now to be detailed.

*Case* 1. Nicholas Landré, aged 55 years, of apparently good constitution, but too much addicted to spirituous potations, and exposed to vicissitudes of temperature, had been about six weeks ill when he entered the Hospital Cochin, on the 3d of April, 1822. For the three last days, his disorder had considerably increased. He had pain under the sternum, extreme sense of oppression—fatiguing cough, which was dry or sanguineous—quick vibrating pulse—pallid countenance—no sleep. He could scarcely walk a step without being breathless. *Bled from the arm.* 4th, In the evening, less oppression. The stethoscope shewed the respiration good on the anterior part of the chest, but not so in the lateral and posterior portion of the right lung. 5th, Return of the oppression—scarcely any respiratory sound in the lower part of the chest. Lies only on his back—expectoration mixed with blood—pulse still hard and quick. 6th, The patient feels strong pulsations in the *right* side. 7th, Was again bled, and experienced a temporary solace. 8th, Oppression greater than ever—prostration of strength—faintness on the least motion—expectoration of fetid and black blood. 10th, A small bleeding was ventured on. 11th, Pain in the præcordial region—extreme anxiety—eyes prominent and haggard. 12th, 13th and 14th. Same state. 15th and 16th, Sense of suffocation—no pulsations to be felt in the radial artery while coughing, the vessel appearing to remain, during that time, tense and full—inability to expectorate. 17th, Discharge of blood from the lungs—great prostration of strength and spirits. In this wretched state he lingered till the 23d, when death put a period to his sufferings.

*Dissection.* A large quantity of turbid serum in the left cavity of the chest, as also false membranes covering the pleura and pericardium. The lungs gorged with a dark-coloured fluid—their tissue black, putrid, and like softened spleen, but still a little crepitous. In the middle of this gangrenous and fetid mass, were seen other masses of *melanose* matters, which, when cut into, presented a solid and smooth surface, with a white nucleus. The larynx, trachea, and bronchia filled with sanguineous and fetid mucus, their lining membrane being of a brownish red colour. The pericardium was red and injected, adhering to the heart by filamentous bands. The heart was rather larger than natural, and the parietes of the left ventricle from six lines to an inch in thickness, of red and compact texture, but the cavity not enlarged—the aortic orifice contracted and rigid.

*Case* 2. Julia Martin, aged 21 years, had not menstruated for two months, and had been ill eight days before she entered the Hospital Cochin, on the 2d of June, 1822. Complains of severe pain in the left side of the chest, particularly during inspiration and coughing. She breathes with difficulty, and spits blood—a crepitous rattling is heard in several parts of the chest. There is fever, a yellow tongue, red at the edges—thirst—inappetency—nausea—vomitings—want of sleep. *Venesection*—15 *leeches to the side of the chest.* No abatement of the symptoms during the succeeding days. On the 11th, prostration of strength—torpor—intense fever—cephalalgia. 13th, To the foregoing symptoms were added a pleuritic pain in the *right* side, to which were applied 30 leeches; without relief—a blister. The symptoms continued during the following days—the patient unable to lie on the right side—the expectoration exhaling a fetid odour. 25th, The anxiety and dyspnœa very distressing. Died on the 26th.

*Dissection.* Dull sound from the chest, especially on the right side, on percussion—the lungs every where adherent to the pectoral parietes by a gellatinous exudation beginning to be organized—some extravasation of turbid serum in the right side. In the lower part of the right lung was found a vast abscess of a gangrenous appearance, ready to burst into the cavity of the pleura. When the horribly fetid contents of the abscess were discharged, the cavity was capable of containing a goose's egg, its internal surface being of a reddish-green colour. The portions of lung immediately contiguous to this abscess were gangrenous. Into the cavity, several bronchial tubes were seen to enter. The rest of the right lung was rather firmer than natural, and a good deal infiltrated with a serous fluid mixed with blood. The left lung was in a state of incipient hepatization—some turbid serum in the pericardium—internal surface of the ventricles of the heart red—nothing particular in the head or abdomen.

*Case* 3. John Fobis, 49 years of age, a baker, of large and robust make, experienced, for the last six weeks, symptoms of pleurisy. He was received in the Cochin on the 10th September, 1822. At this period he suffered pain in the right side of the chest, and, for the five or six preceding days, the cough and sanguineous expectoration had increased—the blood thrown up, (which was in abundance) was liquid and black—oppression extreme—countenance pale, livid, and sunken—skin warm and moist—pulse small, quick—great prostration of strength. The right side of the chest emitted a dull sound on percussion, and was more protuberant and less mobile than the other side, during respiration. On applying the stethoscope, pectoriloquism was immediately perceived, under the angle of the scapula on the right side. M. Cayol pronounced a pleuritic effusion on the right side of the chest. 11th, The odour of the expectoration was insupportably fetid and gangrenous. In this condition the patient survived till the 22d, when death closed the scene.

*Dissection.* More than a pint of turbid serum in the right side of the chest, of horrible odour. Another effusion was found between the base of the lung and the diaphragm, in a kind of cyst formed by adhesions.

Many false membranes in this side of the thorax. The lung itself rather condensed, and little crepitous, especially at the upper part, where it was completely hepatized, and infiltrated with a reddish fluid of gangrenous odour. In the middle of the posterior part of this lung there existed an excavation capable of containing a hen's egg, which was partly empty, and partly filled with a sanious fluid of very fetid odour. This excavation was contiguous to a portion of lung that was actually gangrenous, and infiltrated with a sanious fetid fluid. The mucous membrane of the bronchia on this side was reddened. In the left cavity of the chest there was some serous effusion and false membranes. The bronchial membrane was also red. The heart was sound—the liver was diseased. There was phlogosis of the mucous membrane of the stomach and small intestines.

*Case* 4. Frances Manger, aged 25 years, was received into the Hospital Cochin, on the 25th December, 1821, for a complaint of the chest, which she said she had contracted after her last accouchement, three months previously. She had now oppression, cough, fetid expectoration, flushed face, acute fever. In despite of bleeding, low diet, and evacuations, the malady gained ground. The tongue became dry, the thirst ardent, the skin arid, the prostration of strength extreme, the breath fetid, with vomiting and diarrhœa. She died on the 17th January, 1822.

*Dissection.* The lungs were adherent to the parietes of the chest in all parts by strong organised membranes. The right lung was perfectly crepitous throughout, and sound. The left lung was of the consistence of spleen rather than liver, (splénesé plutôt qu' hepatisé) with here and there some crepitous portions. In the centre of this lung there was a softened portion, of livid colour, and fetid gangrenous odour, forming a kind of depôt of putrid matters, the detritus, apparently, of the lungs. This gangrenous excavation was prolonged by means of a fistulous canal to the third intercostal space, where was found a collection of purulent grumous matter. The pulmonary substance in the neighbourhood of these morbid parts was condensed into a kidney consistence. The liver was enlarged, and of unhealthy structure.

*Observations.* This is a rare case. Had the patient lived some time longer, there would have been an external opening in the intercostal space alluded to, and a communication thus made between the surface and the central depôt.

From the foregoing cases, our author draws the conclusion, that the *general* symptoms of the disease in question are, the fetor of the breath and expectoration, with or without the presence of more or less blood or rather black sanies. There was also in the whole of the four cases, great prostration of strength, and tendency to faintness on any muscular exertion—a smallness and concentration of the pulse—a leaden, pale, or blueish aspect of the countenance—aridity of the skin—symptoms which our author thinks are not merely owing to inflammation of the lungs, but to a "general infection of the fluids, and particularly of the blood—an infection produced, partly by the cause of the gangrene, partly by the resorption of a portion of the matters contained in the gangrenous depôt." It is to this infection of the fluids, he conceives, that the death of the patient is to be attributed, and not to phlogosis. Laennec appears to view things in the same light, and to compare the disease in question with idiopathic gangrenous affections, as anthrax, malignant pustules, pestilential buboes, &c.

---

9. *Epilepsy, with Remarkable State of Pulse.** This is a very curious case, and we shall condense the particulars for the information of our readers.

The subject of it was an officer of the Navy, 46 years of age, who had experienced, sixteen years previous to the date of report, (1820) a single paroxysm of epilepsy, which did not return for 12 years. He then had another attack in bed, and rolled out on the floor. On the 23d August, 1820, Dr. B. visited him, and concluded that his complaint was epilepsy. By proper remedies he was kept free from attacks till January, 1821, when he had several accessions. On the 27th of this month, while Dr. B. was with him, he had four or five paroxysms, some lasting only a few minutes, and not followed by any disposition to sleep. They were commonly preceded by nausea, a sense of aura epileptica rising from the stomach to the head. Moderate bloodletting, purgatives, and light tonics, with pil. hyd. and the tinct. val. ammon. together with a seton in the neck, stopped the attacks for the time. In the beginning of May, Dr. B. again visited the patient, and found that he had had some slight paroxysms. But he complained of great uneasiness and a sense of distention about the epigastrium, which purgatives had failed to remove. He also complained of dyspnœa, and the necessity of sitting upright in bed. On examination, his pulse was found to beat only 36 in the minute, but it was regular and small. On the following morning it was beating only 20 in the minute, and, in the evening, got up to 32. From this time till the 6th May, the pulse varied from 28 to 56 in the minute, but without any return of epilepsy. The patient went to London and had the advice of a physician, which he followed till the 2d July, but without any benefit. On the latter day he was again seen by Dr. Burnett, and complained of great languor, uneasiness, and fulness about the epigastrium. On examination, there was a tenderness with fulness towards the scrobiculus cordis. The pulse was 56 in the minute, and the spirits depressed. He was cupped in the region of the liver, and took aperients. 3d, Had several slight attacks in the night—nausea—pulse 24—tenderness in the region of the liver. To rub in some camphorated mercurial ointment on the left side, and

* Dr. William Burnett, Member of the Royal College of Physicians, Physician in Ordinary to His Royal Highness the Duke of Clarence, one of the Medical Commissioners of the Royal Navy, &c.—*Med. Chir. Transactions. vol.* xiii, *part* I. *p.* 202.

take blue pill with colocynth at night, with decoction of taraxacum in the day. The report on the 9th was, that he has had several slight epileptic seizures, generally occurring after dinner. The pulse is 52, and firmer. 16th, The same in respect to attacks; but he looks thinner, and complains of much fulness and uneasiness about the epigastrium—appetite falling off—pulse 20 in the minute. Infusion of cascarilla with cardamoms and sulphuric ether thrice a day. 17th, Drs. Burnett and Sanden met in consultation. The pulse was 18 in the minute—stools bilious, and the patient thought he felt some mercurial action in his mouth. The mercurials were omitted and the draughts continued, with an aperient. 20th, Had slept pretty well in the first part of the preceding night, but had some paroxysms towards the morning, rather stronger than usual. Pulse 14 in the minute. A blister to the region of the liver—aperients—anodyne at night. 21st, Pulse 14 in the minute. 22d, Passed a very bad night—the paroxysms severer than for a long time past. During the paroxysm the pulse is altogether suspended—the face becomes pale and convulsed—a transient flush then succeeds—the pulse is again felt, and recollection is recovered, to be quickly lost in another attack. The oppression about the præcordia is very great to day—pulse 74 for a minute, then intermits for seven, eight, or ten seconds. In the evening his pulse was at 20. 24th, Pulse from 16 to 18 in the minute. 25th, "The attacks are now more like spasmodic twitchings than epilepsy; but they are very frequent, and the pulse is often suspended for ten or twelve seconds." A draught of black-drop, ammoniated tincture of assafœtida, tincture of cardamoms, and peppermint water, every night. August 2d, No paroxysm since the 25th, but the twitchings continue—sleeps well—pulse 24—tongue clean. Anodyne omitted, and a tonic twice a day. The patient now removed to another part of the country; but Dr. Burnett has heard that the slowness of pulse still continues, with occasional attacks of epilepsy—and also, that he has become affected with anasarcous swellings in different parts of the body.

From the phenomena exhibited by this gentleman, we have little doubt that there is some organic affection of the heart, as well as considerable derangement of the biliary organ. The dropsical swellings may be regarded as a harbinger of bad omen, although they may keep off the fatal event for a greater or less space of time.

Dr. Burnett has found two cases, in the writings of Morgagni, which appear to bear some analogy to the present. The first patient was a priest, who, in his 68th year, was attacked by epilepsy, which left behind it the greatest slowness of pulse, and a coldness of the body. The epilepsy often returned, but the slowness of pulse continued permanent. The first paroxysm was succeeded by pain in the right hypochondrium, which was removed by bilious dejections. Morgagni does not state distinctly the minimum of the pulse in this case, but Dr. B. thinks it did not fall below 24, from an incidental quotation from Gerbezius.

The other case mentioned by the Italian professor, exhibited a similar slowness of the pulse, and was supposed to arise from disorder of the chylopoietic viscera. The case terminated fatally, and many pints of water were found in the thorax, together with various visceral derangements.

We believe, with Dr. Burnett, that the epileptic symptoms, in this case, arose from disorder of the digestive organs. Dr. B. has lately treated a young lady, who had long been subject to severe attacks of epilepsy, by medicines directed to the improvement of the digestive functions, with so much benefit that she has not had a paroxysm for nearly two years.

---

10. *Inflammation of Veins.** In M. Bouillaud's paper, the author proposes to present some new facts calculated to illustrate the history of venous inflammation, and then, to draw from them some general conclusions. It is with the facts we have first to do.

*Case* 1. Aubart, 21 years of age, was brought to the Hospital Cochin, on the 8th November, 1822, in a state of prostration and delirium that prevented her giving any history of her disease. At that time, her lips and teeth were covered with a black sordes—face pale—tongue dry and rough as a file—thirst ardent—pain and swelling of the parotids—abdomen tender on pressure—high fever and intense heat of skin—pulse 140—delirium loquax—subsultus tendinum—picking of the bed-clothes—frequent cough. The fever continued the three following days, with evening exacerbations. During the fourth, fifth, and sixth days, the fever subsided, and the patient became sensible, craving for food—but this did not continue; she relapsed again, and died on the ninth day from her entrance into the hospital.

*Dissection.* The lungs were crepitous—adhesions between the pleuræ—mucous membrane injected—considerable quantity of bloody serum in the pericardium—heart rather flaccid—the internal surfaces of the cavities of the heart, especially the right chambers, were red—the aortic valves, and the inner surfaces of the aorta and its trunks, were like scarlet—not apparently from injection of the vasa vasorum, but as if they had been dyed with a red tincture. The pulmonary artery and its valves presented a similar tint, but much fainter. The internal surface of the venous system generally exhibited a deep brownish-red colour. The deep-seated veins of one lower extremity (which had become infiltrated before death) were obstructed by a long fibrinous concretion, extending to their junction with the inferior cava—the veins of the other limb contained fluid blood—the stomach and bowels presented nothing very particular externally—but their mucous membrane was highly coloured—and ulcerations were found in that of the small and large intestines—especially the ileum and colon. The liver and spleen were

---

* 1. Recherches Cliniques pour servir à l'Histoire de la Phlebite. Par M. J. Bouillaud, M.D. Revue Med. Avril et Juin, 1825.

2. Exposé succinct des Recherches faites sur la Phlebite. Par M. F. Ribes, M.D. Revue Med. Juillet, 1825.

enlarged. The meninges of the brain were a little infiltrated, with some serous effusion at the basis and in the ventricles.

*Remarks.* "We see in this case all the symptoms of adynamic or putrid fever, coinciding with inflammation of the blood-vessels; but, as there existed, at the same time, a phlegmasia of the mucous membrane of the digestive tube, some doubt must arise as to which of the inflammations was the primary one, and the cause of the fever. Some people would not hesitate a moment to place the fever to the account of the gastro-enteritis; but, what weight are we to attach to such opinions, after shewing, on various occasions, that all the phenomena of fever may take place without the presence of any gastro-enteritis at all?"

*Case* 2. A young woman, aged 20, of sanguine temperament, accustomed to hard work in the country, and often affected with pectoral complaints, experienced a suppression of the menses, in consequence of a fright, soon after which she felt oppression, with cough and general malaise. On the second day, fever preceded by rigor. Having taken some hot wine, she was seized with violent cholic and vomiting. In the evening there was an exasperation of the symptoms, and the patient complained of acute pain in the right side, extending to the arm-pit—augmented by coughing—the expectoration being tinged with blood. She was bled from the arm, and, being very fat, several punctures of the lancet were made before the blood would freely flow. These manœuvres caused great pain at the time. In the evening of the day she was bled, the pain extended to the arm-pit, and the lancet-wound was swelled and inflamed. Another venesection, in the same arm, was practised on the 5th day, but the fever and pulmonic symptoms increased—the arm became very painful and swelled, with an erysipelatous appearance, a reddish ichor oozing from the lancet-wound. On the 6th day, the veins could be felt like hard cords extending up the arm, and the least pressure or motion excited acute pain. The pleuro-pneumony still continuing, the patient was bled from the other arm, but without benefit. Inflammation quickly appeared around the new orifice, with pain and swelling of the arm. The symptoms now became graver and graver. The restlessness was extreme—the expectoration black—sleep entirely gone. 8th day, Delirium, which augmented in the evening to fury. 9th day, Death.

*Dissection.* All the veins of the right arm (the first bled) were red and thickened, and contained a purulent and sanguineous fluid of fetid odour. Several small abscesses were scattered about in the neighbouring cellular membrane. The glands of the axilla were enormously enlarged. The mucous membrane of the larynx, trachea, and bronchia, was red, and covered with mucosities. The right lung was hepatised at the upper part, and exhaled a gangrenous odour. The bronchial glands were gorged and black. The heart and arteries were sound, as were also the great venous trunks. The membranes of the brain were inflamed, and there was some effusion into the ventricles.

*Case 3.* A man was received into LA CHARITÉ in the beginning of January, for a catarrhal complaint, which gave way to the common means. On the 24th of the same month, when preparing to quit the hospital, he was suddenly seized with difficulty of breathing, high fever, pain in the right side, which quickly shifted to the left. On the 25th, the pain seemed fixed in the region of the heart. The chest sounded well throughout; but still the breathing was short and laborious. *Venesection—30 leeches to the left side.* 26th, The pulse strong as ever —the respiration short—little cough, no expectoration. An erysipelas became developed at the bend of the arm, where the vein was cut. Another venesection was performed in the same place. The patient died next morning at 5 o'clock, without delirium or any failure of the intellectual functions.

*Dissection.* The cellular tissue of the arm affected with erysipelas was infiltrated. The heart was of its natural size, and the left chambers presented nothing worthy of remark. The lining membrane of the right chambers was manifestly inflamed and quite red. The inflammation extended to the two cavæ, to the jugulars, to the veins of the arm, particularly the arm that was bled, and even to the crural veins. The abdominal aorta presented, here and there, some patches of insulated redness. There was no other appearance that deserved notice.

*Remarks.* As no organ in the body presented lesions sufficient to account for the death of the patient, our author thinks himself justified in attributing it to the inflammation of the venous system, and to the consequent alteration in the circulating fluid itself. Our author makes some animadversions again on the new physiological doctrine, which we shall pass over, as we have sufficiently examined the doctrine of the school of Broussais in another place.

*Case 4.* The fourth case was that of a man who had one of his legs crushed to a mummy by the fall of a stone upon it. For reasons not mentioned, amputation was not performed on the spot, and it was too late after inflammation had commenced. The poor man died of mortification of the member, and the veins were found inflamed as high as the heart. There was also inflammation of the internal tunic of the aorta and its primary branches. There was great alteration in the circulating fluid, and a considerable extrication of gas in the venæ cavæ. We adverted to this case chiefly to shew the necessity of *immediate amputation,* in cases where there is no chance of saving the limb, as was the case in the present instance. There is every probability that this man's life might have been saved, had the jammed member been removed immediately after the first shock of the accident was overcome, and before re-action, or, at least, inflammation was set up.

*Case 5.* Early in September, 1824, a man was conveyed to LA CHARITÉ, who had had a whitlow on the fore-finger of the left hand for five or six days. M. Roux had made a free incision into the part, and the man was put on rigorous abstemiousness. The first and second days passed without any thing particular; but, on the third and fourth, there arose alarming pain, with swelling and œdema—and, ultimately, gangrene to a considerable extent. There was great head-ache and fever. M. Roux proposed amputation, which was not acceded to by the patient. On the fifth day there was great prostration—feeble pulse—stupor—black tongue—swelling of the arm, &c. These symptoms continued, and indeed increased, till the seventh day, when death closed the scene.

*Dissection.* The veins of the arm, the axillary, subclavian, jugular, and other veins were found inflamed. They were empty of blood, and their internal surface covered with false membranes and a layer of puriform matter. There was no disease in the chest or abdomen.

The three remaining cases were more or less complicated, there being not only inflammation of veins but of other organs, rendering it sometimes difficult to determine which was entitled to priority, and which was the cause of death. There is one of these cases, however, which is worthy of notice, in a surgical point of view.

*Case* 6. Francis Badaud, entered La Pitié on the 11th September 1824, having a varicose ulcer on the leg. M. Lisfranc divided the internal saphena vein on the 13th September. During the two succeeding days there was no pain nor inflammation. 16th, Pain in the wound. 17th, Pain was propagated along the track of the vein. 18th, Decided phlebitis was developed, with fever, and red tongue. *Twenty-five leeches to the part.* 19th, The sphere of the phlebitis increased—20 *leeches applied.* 20th, The pain on the inside of the leg very severe—30 *leeches applied.* 22d, There were symptoms of slight gastritis, and 25 *leeches were applied to the epigastrium.* 23d, The pain was very severe in the course of the saphena vein, the tongue was very red and dry—the fever considerable. *Thirty leeches to the epigastrium.* Symptoms of adynamic or low fever now became developed, and death took place on the 25th of the month.

*Dissection.* The veins of the leg, thigh, and pelvis were found to be red, and thickened in their coats. There was decided inflammation of the stomach, and also of the small intestines.

*Remarks.* There can be little doubt, we think, that the operation in this case was the cause of the phlebitis, and that the venous inflammation preceded the gastric.

*Anatomical Characters of Phlebitis.* These vary according to the violence and the duration of the inflammation. In the early stage, we find the internal membrane of the vessel of a brownish-red colour, with or without traces of vascular injection. At a more advanced period, this membrane becomes thickened, and easily lacerated. In this condition, it is readily separated from the subjacent tunic, and is often covered with purulent matter, or ulcerated in its structure to a greater or less extent. It is still more common to find the pus, which is the product of the inflammation, mixed with a certain quantity of the blood contained in the vein—hence the purulent, sanguinolent, or fetid matter which we see in the vessels of many of our sick—hence the manifest decomposition of the blood, with the presence of gas, which we occasionally observe. In some cases, the purulent matter secreted by the inflamed vein, determines a sort of coagulation of the venous blood, and produces those long fibrinous clots, which obstruct the canals of the vessels. Sometimes, though rarely, the concretible and organisable matter secreted by the vein agglutinates the opposite parietes, and the vessel is ultimately converted into a solid cord. This, however, is a rare circumstance, because the matter, as it is secreted, is hurried along into the general circulation. When the inflammation has, at length, invaded all the coats of the vein, the vessel becomes thickened in its parietes, and readily lacerable.

Such are the alterations resulting from acute phlebitis. When the disease has passed into a chronic stage, it gives birth to fibrous, fibro-cartilaginous, or even calcareous indurations, which it is not very unusual to meet with in the parietes of these tubes. "It is," says our author, "to the same cause, (chronic inflammation of the internal membrane of the venous system) that we are to attribute the induration of the tricuspid valve, with more or less narrowing of the auriculo-ventricular opening of that side.*

*Etiology.* Among the most common causes of phlebitis, must be ranged those injuries to which the veins are exposed in various operations and accidental lesions. The writings of Hodgson and Breschet sufficiently attest the extent of this etiological list. There are some other causes also. Hunter has seen phlebitis succeed gangrene. It has been often observed in puerperal fever. Our author has frequently seen phlebitis developed in people who had sunk in bad fevers—and in very many instances of great local inflammation, which seemed to have been gradually propagated to the general vascular system. "The introduction of matters more or less acrid and irritating into the venous system, is a cause of phlebitis not sufficiently explored, but which well merits attention. I am persuaded, that to this cause, is due the venous inflammation, which I have so often seen in the bodies of patients who died of malignant fevers—for all these fevers depend on a greater or less alteration—on a kind of *disorganization* of the blood, complicated

* This is by no means improbable. When we consider the incalculable quantity and number of deleterious substances introduced into the circulation through the medium of food and physic, it is really wonderful that the conduits of the blood are not more frequently affected than they appear to be. We see more readily the effects of those noxious agents on the digestive organs, to which they are first applied; but we cannot doubt that they produce also an ulterior effect very often on the finer channels through which they pass in the processes of assimilation, sanguification, secretion, &c These channels are seldom explored in our anatomico-pathological researches.

with a general phlegmasia produced by deleterious matters mixing with the current of the circulation."

*Symptoms.* 1. The symptoms of inflammation in the trunk of a superficial or external vein are easily recognized. The member swells, becomes hot, painful, or is even the seat of phlegmonous erysipelas. The vessel itself feels tense, hard, knotty, or like a cord. Abscesses not unfrequently form in the course of the vein. The pain, our author thinks, is more dependent on an affection of the neighbouring nerves, than on inflammation of the vein itself. Œdema of the limb is a very common attendant on phlebitis of one or more of the principal veins, and evidently arises from the mechanical obstruction to the return of the blood—the veins being now acknowledged to be the principal conductors of the serous exhalations that take place into the cellular tissue. Such are the signs of local phlebitis.

2. When the inflammation extends to the whole, or to a great portion of that vast membrane which lines the internal surface of the venous system, we constantly find that a violent fever is lighted up. "Among many of our patients, the fever presented all those characters which are attributed to what are called putrid adynamic, or typhoid fevers. And, indeed, the term *putrid* is perfectly applicable, since after, nay *before* death, there are unequivocal signs of decomposition, or a kind of *putrid fermentation* of the fluids. The case No. 4, exhibited these phenomena." Our author is not the only nor the first person who has observed this phenomenon. "When the inflammation," says Mr. Hodgson, "is propagated to the principal trunks of veins, and when there is pus secreted in the vessel, it is accompanied by a very intense constitutional irritation, and symptoms which bear the strongest resemblance to those of typhoid fever." Mr. H. relates cases in proof of this assertion. The same remark has been made by Mr. John Hunter. Dr. Breschet in his excellent Memoir on Phlebitis, expresses himself thus:—"Many physicians, in cases of venous inflammation, have observed the phenomena proper to typhus fever; and I myself have repeatedly discovered evident marks of inflammation in the veins and sinuses of the encephalon in those who have died of typhus fever." But although many physicians have observed these phenomena, none of them have attempted an explanation of them. Our author ventures to fill up this lacuna.

He begs the reader, in the first place, to recollect that, when deleterious or putrid matters, such as pus, urine, &c. are injected into the veins of animals, an artificial fever is raised exactly like that denominated putrid or typhoid. The illustrious Baglivi *infused into or inoculated*, if we may use the expression, several animals with fever, by injecting into their veins acrid, spirituous, and irritating substances; and in our own times, Magendie and Gaspard have done the same thing, by means of putrid injections. If we reflect a little, we shall acknowledge that the individuals who are affected with venous inflammation of considerable extent, are under circumstances extremely analogous to those in which the animals, thus experimented on, are placed. We need not, therefore, be surprised if phlebitis is often accompanied by all the phenomena characteristic of putrid or adynamic fevers.

And here, while speaking of fever, our author craves permission to say a word or two in regard to the celebrated Broussais. "Every body knows that this eminent pathologist considers gastro-enteritis, as the cause of all those fevers called *idiopathic* by systematic writers, and that he does not admit the existence of general diseases. The observations and cases which are here stated, are not very favourable to this doctrine. We have related several cases of putrid or adynamic fevers, where nothing like gastro-intestinal inflammation could be detected; and we have cited several instances where fevers of the above character were artificially produced by the injection of putrid matters into the veins. From this it appears evident, *first*, that gastro-enteritis is not essential to the existence of fever—and *secondly*, it would appear probable, at least, that these fevers depend on a general inflammation of the sanguiferous system, attended with more or less alteration of the blood and other fluids." This double conclusion appears to M. Bouillaud incontestible—and he appeals to M. Broussais himself for its legitimacy.

*Treatment.* Partial phlebitis is to be treated like any other local inflammation by bleeding, low diet, and diluents. General phlebitis also requires the antiphlogistic plan of treatment. But then there is another grand and fundamental indication to be fulfilled, if possible—namely, to counteract the alteration of the blood, vitiated as it must be, by the admixture of pus secreted by the inflamed vessel. Hitherto, this indication has been entirely neglected, and the patient has been consigned to an almost inevitable death, when affected with general phlebitis and secretion of pus. Time may bring to light some means of counteracting this pathological condition, but at present, we are obliged to trust almost exclusively to the efforts of Nature, in bringing about a salutary crisis.

M. Bouillaud terminates his Memoir with the narrative of a few cases of partial phlebitis cured by the antiphlogistic plan, but these cases need not here be detailed, since the plan is well understood in this country.

We now come to the Memoir of M. Ribes, a gentleman of great talent and accurate observation. In the year 1816, M. Ribes published a summary exposition of the observations which, in the course of his pathological researches, he had made on the subject of venous inflammation. He was afterwards on the point of publishing a more extended memoir on the same subject, when a translation of Mr. Hodgson's work, with notes by Breschet, prevented him for that time. Nevertheless, he conceives, that some of his facts and conclusions may not be unworthy of the attention of his professional brethren, when given in a very succinct and condensed form. These facts and conclusions we shall still farther abbreviate, but, we hope, without omitting any thing that is useful or interesting.

1. When we examine the veins, in the dead subject, after emptying them of blood, we observe, in the majority of instances, that their parietes are pale, and without any variation of hue, except that the small ramifications are, by their nature, almost transparent. In some bodies, however, we perceive that the veins containing fluid or coagulated blood, are, in some places, tinged, and in others, of their natural colour. How is this? The tissue of the vein must have experienced some alteration, either from the effect of disease, or the natural consequence of death, otherwise it would present a uniformity of aspect. It is certain that the tinged portion of vein, which is sometimes of the colour of lees of wine, is not ordinarily rendered so by disease. Inflammation leaves effects of a different kind—but sometimes it is not very easy to tell the one from the other.

2. The veins are very frequently inflamed; and this affection is a very dangerous one. These vessels become inflamed in various degrees, and then present various appearances. Our author has seen a great number of veins inflamed cotemporaneously in the same individual. The small veins have appeared to him to be more subject to this disease than the large. He is convinced that erysipelas has its principal seat in the venous capillaries. He has also found the large veins, as the saphena, tibial, femoral, deep-seated veins of the arm, but more especially the superficial veins of the upper extremity, very frequently inflamed. The veins of the abdomen are very subject to this affection, as are the inferior cava and its branches; but the vena portæ and its branches are more prone to inflammation than any other veins of the abdomen. The sinuses of the dura mater, very frequently present traces of phlogosis. He has seen the inflammation bounded to a small space, and also extending in all degrees, even up to the auricles of the heart. In the first grade, or early period of phlebitis, the vasa vasorum of the internal tunic are merely gorged with blood, and the interstices between these small vessels are pale:—but when the phlogosis is more advanced, the whole surface of the part becomes red. This redness is very different from that of lees of wine already alluded to, and which appears to be a mere tinge from the contained blood. In phlogosis, the colour is seen to evidently depend on the blood contained in the gorged vasa vasorum, which have a reticulated disposition. The other tunics of the vein are successively invaded by the inflammation, until the three coats appear homogeneously phlogosed. At this period, the internal tunic becomes thickened—so much so, sometimes, if the inflammation has been intense, that the calibre of the vessel remains open and circular, when it is cut across.

3. In examining inflamed veins, several different changes of structure become apparent. Sometimes their parietes are smooth, sometimes rough, ulcerated, and covered with villosities. Occasionally, our author has seen a false membrane, like that which takes place in croup, thrown out on the internal surface of an inflamed vein. He has seen these false membranes of various consistencies—some of them approaching to organization. A remarkable preparation of this kind was lately shewn to our author by M. Chaussier. It was taken from a woman, in whom the left renal vein, and also the vena cava, as high as the diaphragm, and as low as the division of the iliacs, were lined with a false membrane, adhering intimately in some places, and very slightly in others.

4. Pus is often found in inflamed veins, sometimes mixed with blood, at other times white, thick, and very distinct from the circulating fluid. Pus, however, is sometimes found in veins where there is no trace of inflammation. In such cases, it must be brought there by absorption, like other substances, as oil, which we occasionally discover in the veins.

5. Sometimes, when a vein is slightly inflamed, it becomes dilated and distended with blood. The blood then stagnates in the vessel, coagulates, hardens, and becomes closely adherent to its internal parietes. The member then generally swells. "But," says M. Ribes, "I have remarked in some individuals, that this swelling had a peculiar character. The parts were of their natural colour—the limb was soft, shining, and did not present any pitting after pressure from the finger, nor any crepitation. Hence the swelling could not be the effect of infiltration, nor the extrication of gas, but a general expansion of all the soft parts of which the member was composed."

6. Our author has seen phlebitis often accompanying varices—and still more frequently resulting from varicose ulcerations, and also from ulcers attended with caries of the bones. Phlebitis was a common attendant on hospital gangrene. M. Ribes has, he says, invariably found the veins inflamed in that peculiar and fatal affection, termed dry or white gangrene of old people. "In erysipelas the veins are inflamed; and the result of numerous examinations which I have made, is a conviction, that the essential seat of erysipelas is in the capillary veins." In puerperal peritonitis, the veins of the abdomen, and more especially of the uterus, will be found inflamed. "In people who have died of low (adynamic) fevers, I have found all the ramifications of the vena portæ unequivocally inflamed."

7. In incipient phlebitis, the patient experiences a slight pain in the track of the veins affected. These vessels gradually swell and become prominent, presenting a light blueish colour, and subsequently a brownish pale hue. The circulation ceases in the vessel, and the blood becomes more or less decomposed. If the circulation should be re-established, the contents of the vein are carried into the current of the circulation, and dangerous consequences may result.

The following are the prominent appearances which were found on dissection, in the bodies of those who have died of phlebitis.

8. The abdomen swelled. (meteorisé)—The stomach and intestines more or less inflamed, especially the mucous membrane. In almost all cases, there were traces of inflammation of the pleura, of the brain or its membranes, with effusion of serum, and other marks of phlogosis.

9. Phlebitis is a serious malady, and is often quickly mortal. Sometimes, however, it runs a long course. Our author has known it continue 40, 45, or 50 days. If phlebitis be situated in vessels at a great distance from the heart, and if the inflammation is bounded to a small compass, there is little danger. If, on the contrary, the inflammation spreads towards the interior, or if it have its primitive seat near the centre of the circulation, the termination is generally fatal.

We shall now present our readers with a condensed account of some of the cases brought forward by M. Ribes.

*Case* 1. This was a very remarkable one, and occurred so far back as the year, 1799. A lady, 36 years of age, had been subject to chilblains of her hands every winter, from which she suffered very much. Our author was called to this patient on the 18th December, and was surprised to find the veins of the arm greatly dilated, and appearing to be filled with very black blood. These vessels were so extremely sensible, that the least pressure caused the patient to cry out. The hand and fore-arm were much swelled, and presented spots like what we see in cases of sea-scurvy. The tongue was not furred, there was little appetite, no thirst, bowels regular, pulse calm in the mornings, but a little feverish in the evenings. Some trifling and inert treatment was ordered, and the patient continued in much the same state till the 25th December, when the pain suddenly became exasperated. 26th, Our author found the patient in great suffering and distress. The pulse being quick and hard, he drew, with great fear, about eight ounces of blood from the right arm, a few hours after which, the patient was much relieved, and passed the night quietly. Things continued the same till the 30th, when the pains became more violent than ever. A poultice was applied to the arm. *2d January*, The patient was found in a state of great restlessness. She had chills and hot flushes. The veins of the arm were found to be red, inflamed, and without a current of blood through them. The pulse was very frequent and irregular—the breathing rather laborious. *3d*, There were appearances of gangrene in the skin of the arm, and the phlebitis had extended higher up towards the trunk. The hand was cold, little sensible, and the circulation apparently arrested. The pulse was now very small, and still intermittent. There was some tendency to drowsiness, which continued during the succeeding days. *8th*, The breathing was very laborious, and there was slight delirium. The gangrene made progress—hiccup became troublesome. Stupor came on, and the patient dragged out a wretched existence till the morning of the 13th January, when death closed the scene.

*Dissection*. The veins of the arm were found highly inflamed. The median and the cephalic veins were extremely dilated, and filled with a purulent matter, which, in some places, was of the colour of the lees of wine, in others, like common pus. The parietes of the vessels were very much thickened, and when cut across, the lumen or bore of the vessels remained circular and open like that of a strong artery. The interior surfaces were ulcerated, and covered with villosities. The axillary vein contained pus, and presented traces of inflammation which were continued into the vena cava, and even into the right auricle and ventricle. There was some serum in the cavity of the pericardium, and also in the left cavity of the chest, the pleura exhibiting marks of inflammation. The mucous membrane of the stomach and intestines was unequivocally inflamed. The tunica arachnoides was thickened and opake—the vessels of the pia mater were gorged with blood, and there was some effusion between the membranes. The ventricles were filled with a bloody serum which was found throughout the spinal canal. The arm was taken on the day of dissection to M. Le Professeur Chaussier, who delivered a clinical lecture on the disease, at *L'Ecole de Medecine.*

*Case* 2. Flicoteau, a military invalid, 55 years of age, had been wounded in the lower part of the left leg by the bursting of a shell. The sore was long in healing, but at length completely closed, and the man enjoyed good health for some years, when, without any ostensible cause, he fell into a state of melancholy and of strange unpleasant feelings, of which he could not give a description. About a month after this hypochondriacal state had commenced, he complained of pain along the inner side of the limb formerly wounded, and on examining this limb, our author perceived that the internal saphena vein was red, painful, and evidently inflamed to the groin. When the vessel was touched with the finger, the patient expressed great pain, but the blood circulated freely through it. The old cicatrix had become red, painful and swelled. A small gangrenous point shewed itself on the inner side of the leg, and spread rapidly—the abdomen became painful and distended—the breathing difficult—the pulse intermittent, and death took place on the 15th day of the disease.

*Dissection.* The arteries of the gangrened limb were in their natural state—the parietes of the veins of the leg and thigh were very much thickened, so as to form a contrast with the large trunks in the abdomen. The saphena, from the ankle to the knee, was red and very much inflamed, containing pus, some of which was yellow, and other portions of a sanguineous appearance. The lymphatics about the groin were very much dilated, but did not contain any liquid. The intestines were inflamed in several places. The other viscera of the abdomen, and those of the chest were sound. There was an abscess discovered under the pectoral muscle, " not apparently a local affection, but a deposit of purulent matter there." In the upper part of one of the lungs there was also another small depôt of matter—not appearing to be the result of local inflammation, but a metastasis of pus from the inflamed vein. The pia mater was slightly inflamed, and there was some effusion into the lateral ventricles. The brain was firm in consistence. We shall conclude with some particulars of one more case.

*Case* 3. A personage of great distinction, aged 45 years, experienced profound and constant pain in the upper part of the head, which reduced him to a state of melancholy and dejection of mind. He was also subject to attacks of epilepsy, at longer or shorter intervals. His intellects for some years, were slightly deranged, but were once more in a state of integrity, when he was suddenly taken with raging insanity, which continued for 25 days, and then subsided. Six months after this event he died, still having the pains in the head, and the dejection of mind till the last. The epileptic paroxysms had also become more frequent than ever. Among those consulted in this case were

Portal, Chaussier, Laennec, Boyer, and other Lions of the French metropolis.

*Dissection.* The cranium in its anterior part was exceedingly thin, hard, without diploe, and transparent. The posterior part of the skull, on the other hand, was remarkably thick and spongy. The internal surface of the skull was covered with asperities and with some remarkable depressions, where were lodged varicose veins. The parietes of the superior longitudinal sinus were very much thickened, and in the anterior portion of this sinus was lodged a fibrous body, two inches in length, flattened and well organised. It was so tough as not to be torn by the dissecting forceps. It was evident that the circulation had ceased in this part for a certain length of time. The posterior portion of the superior longitudinal sinus was very much enlarged, being an inch in circumference, and filled with black coagulated blood, surrounded by a crust of fibrinous matter. The thickened parietes of the sinus were reddened, and exhibited unequivocal marks of inflammation. This sinus was lined throughout with a false membrane, the natural result and sure criterion of phlogosis. From this sinus went off an enormously enlarged varicose vein, which was lodged in a deep gutter of the parietal bone, and was filled with a clot of grumous blood. The right lateral sinus was free—the left was blocked up by an organised fibrous mass, so that no blood could pass in that direction. The membranes of the brain were unequivocally inflamed, and there was much fluid infiltrated between them, and also into the lateral ventricles. The left hemisphere of the brain was of its natural consistence—the *right* was softened, and of a yellowish colour. Numerous bloody points presented themselves on slicing the brain. In the centre of this (the right) hemisphere, was developed an ovoid tubercle, the size of a pigeon's egg, which projected a little into the ventricle of that side. This tubercle increased in density as its centre was approached, and this centre was composed of a vesicle with dense parietes, inclosing a viscid fluid of the colour and consistence of bile. "This tubercle," says our author, "which was evidently of a *cancerous nature* (!) was undoubtedly the cause of all the cerebral disorders."

M. Ribes concludes his memoir with some observations on the new physiological doctrine, to which he cannot subscribe. He too has endeavoured to trace the cause of adynamic, or idiopathic fevers. He first examined the solar plexus and the nerves emanating from that abdominal brain, but could make out nothing satisfactory. He then examined the cœliac and mesenteric arteries of those who died of idiopathic fever, but with no better success. He lastly investigated the state of the venous system in such subjects, and the following was the result.

"In almost every person who died of adynamic fever, I found traces of inflammation in the trunk and branches of the vena portæ, and sometimes even in the cavæ hepaticæ, up to the right auricle of the heart. I found this to be the case in so many instances that, in the year 1816, I announced it as highly probable that the venous system was the part principally affected in adynamic fevers. It is true that we shall meet with individuals where the traces of inflammation are very slightly marked; but we know how rapidly these traces sometimes disappear in the dead subject. If we examine the seat of erysipelas in a subject dead of this disease, almost every vestige of the inflammation will have disappeared. It is the same with inflammation of the veins; but, however slight the phlebitis may have been, the eye experienced in these investigations will generally detect the proofs of its existence."

Thus, we see that a new seat of fever is now to be admitted. One thing appears clear to us, namely, that every new seat of fever that is discovered is a fresh proof that fever has no exclusive seat at all. Every local inflammation will produce the phenomena of fever; and every idiopathic fever will occasionally give rise to local inflammation. This, we believe, is the true state of the case.

We think the present investigation of phlebitis is highly deserving of the attention of the profession, as enlarging our views of diseases, their seats, and their causes.

---

11. *Perforation of the Stomach.* Mr. Barber, (of Henrietta-street, Covent-garden) a gentleman of middle age, had been affected with daily vomiting for nearly 20 years; and his father had been subject to the same complaint, of which he ultimately died. Mr. Barber, however, was rarely prevented from attending to his usual concerns, up to the day before his death. The vomiting generally came on some two or three hours after eating, and blood was frequently seen in the ejected matters. He occasionally complained of pain in the region of the stomach. He was not at all emaciated—but, on the contrary, was rather fleshy. On Tuesday, the 6th December, 1825, he ate a hearty dinner of beef-steaks and drank some spirits and water afterwards. While in this last act, he was seized with sudden and violent pain in the region of the stomach, which soon spread all over the abdomen, attended with constant restlessness, quick pulse, anxious countenance, and tendency to sickness. Mr. Best, surgeon, of Tavistock-street, was called to the patient, and bled him, directing such other measures as he deemed proper. At one o'clock, p. m. on Thursday, the 7th, Dr. Johnson was called in, and found the patient with the symptoms above-described, the abdomen being very tense and distended, the pulse 120 and sharp—the tongue coated—the countenance indicative of the greatest distress. On examination, it was perceived that an old inguinal hernia, which had lately given no uneasiness, was moderately distended, and its contents easily reducible; but these contents immediately returned, as soon as pressure was taken off. Dr. J. suspecting that there might be some strangulation about the inner ring, suggested the propriety of an operating surgeon being joined in the consultation, and Mr. Stanly attended at 4 o'clock. Meantime the abdomen was covered with leeches, and attempts were made to open the bowels, both by medicines and enemata. Mr. Stanly examined the hernia, but could not perceive that there was any proof of strangulation. The medical attendants met again at 10 o'clock, and it was evident that the patient was at the point of death. He had vomited upwards of three pints of a black fluid, resembling coffee grounds. The pulse was scarcely perceptible, and the countenance was ghastly. The hernial tumour was now larger than ever, and a crackling or gurgling noise was heard in it when pressed up towards the abdomen. Mr. Stanly now thought it advisable to proceed to an operation, in order to ascertain, more exactly, the state of the hernia, giving the friends but little hopes of success at the same time. After laying bare the sac, which was much distended, Mr. S. continued to divide layer after layer, with great caution, when suddenly, on making a small puncture, a rush of air, followed by a discharge of reddish fluid, took place. At each inspiration these fluids were discharged, in considerable quantities, and with a gurgling noise—and these discharges continued without any abatement, so that it was evident a communication had been made with the abdomen, but whether with the internal surface of the bowels or with the peritoneal cavity, was not certain. Under these circumstances, and, especially, as the patient was evidently dying, it was not deemed prudent to proceed farther with the operation. A ligature was passed round the puncture, the lips of the wound stitched, and adhesive straps applied. At half-past 11 Mr. B. died.

*Dissection.* The stomach was of a most enormous size, and its peritoneal coat, together with the whole peritoneal covering of the intestines greatly inflamed. A quantity of dark-coloured fluid, nearly resembling that which the patient had vomited, was found diffused in the general cavity of the abdomen, in which there was also a good deal of air, which escaped on first opening the cavity. Near the pyloric orifice of the stomach, a small opening, the size of a silver penny, was discovered externally. The viscus was then cut out carefully, and examined. On its internal surface, and corresponding with the aperture discovered externally, there was an old ulcer, with hard, white, and raised edges. In its centre was the aperture abovementioned. No intestine or omentum was in the hernial sac, which was filled with the same kind of fluid as that contained in the general cavity of the abdomen.

The whole of the phenomena were now clearly accounted for. The ulcer in the stomach had existed for years, and gave origin to the vomitings and bloody discharges. On Tuesday the bottom of the ulcer gave way, and the contents of the stomach were discharged into the abdominal cavity—hence the pain, inflammation, and distention. Part of this extravasation (both fluid and aerial) had made its way into the hernial sac, and thus caused the puzzling phenomena above-mentioned, in the operation.

It is a curious circumstance that Mr. Barber's father died, apparently of the same disease, but no post-mortem examination took place. This event led to a ready permission, on the part of the relatives, for opening the body, of which the medical attendants quickly availed themselves.

12. *Hydrophobia.** There can be no doubt that a train of phenomena closely resembling rabies, or hydrophobia canina, may arise without any bite of a rabid animal. Those, indeed, who have seen much of tetanus, must have observed cases of this disease, where the dread and the difficulty of swallowing liquids were nearly, if not quite as great as in hyd. canina. The case related by Dr. Whymper, in our cotemporary, does not, therefore, excite much surprise, though it is far from impossible that the unhappy patient had received the poison of a rabid animal previously. We know that many months often elapse between the bite and the hydrophobia, in which interval a man might readily forget the circumstance;—nor would any trace of the wound remain.

*Case.* A robust soldier plunged into a river after being heated much in the sun, and while perspiring profusely. In the night he felt chilly and unwell, and towards the morning, "he complained of a sense of weight on the upper part of the thorax of the left side," with a benumbed aching pain down the left arm. He was conveyed to the hospital in Dublin, under Dr. W's. care. He had now extreme anxiety of countenance, irritability of manner, complained of some pain, and great oppression under the five upper ribs of the left side, with obtuse pain in the shoulder and arm, pulse oppressed and wiry, with irregularity and intermissions, breathing slow and laboured, much thirst, yet resistance to drink, as he said the attempt took away his breath. He was bled, had a warm bath, and was purged freely, by which means he was much relieved. Next morning he was worse than ever. He, this day, in short, presented the usual symptoms of hydrophobia, which continued about 30 hours, when a state of rapid collapse succeeded. The nervous excitability suddenly ceased—the respiration became natural—"he swallowed whatever was given him with ease." He sunk into a state of syncope, and expired.

On dissection, the muscular structure of the heart was found to be so flaccid that it could be "torn as easily as soaked pasteboard." This was almost the only deviation from a natural state which could be found.

The disease, in some of its phenomena, and especially in this state of the heart, resembled angina pectoris. While, on the subject of hydrophobia, we would suggest to our brethren the propriety of putting in practice, in all cases of bites from suspected animals, the means recommended by Dr. Barry—namely, the application of cupping-glasses over the part, which should be kept on for an hour or more, and then the part cut out, after which, the actual cautery should be applied, so as to hermetically seal the mouths of the vessels. By these means, Dr. Barry rescued animals from the effects of poisons introduced into wounds, even after part of the poison had made its way into the torrent of the circulation. It is curious that the same plan was recommended by Celsus—and even before his time, the practice of sucking poisoned

* Dr. Whymper. Med, and Phys. Journal, November, 1825.

wounds was common among many nations. We shall give a more detailed account of Dr. Barry's experiments in another place.

Dr. Whymper appends the case of a man who attended on the patient abovementioned, and who appears to have experienced some powerful moral impressions from the fatal scene which he witnessed. He was admitted into hospital a short time after the event described, presenting nearly the same symptoms, but in a much milder degree. He had a great dislike to drink, as he said it took away his breath. He was twice bled to *deliquium*, and had strong purgatives. He recovered, and left the hospital; but was soon obliged to return. "He has still a remarkable agitation of manner and hesitation of speech. The *pulmonic* disorder under which he labours is constant dyspnœa, palpitation, with occasional attacks of asthmatic paroxysms." We venture to say that Dr. Whymper's patient labours under *cardiac* rather than *pulmonic* disorder. The phenomena *appear* to be in the organs of respiration—the cause is in the heart. Why does not Dr. W. investigate the chest by auscultation and percussion? Surely such means are not neglected in the sister isle. This case (no very infrequent one) appears to have arisen from powerful moral emotions deranging the functions of the heart and respiratory organs, and now settling into incurable disease. It is in corroboration of the pathology which we alluded to in the first case.

---

13. *Globus Antiperistalticus.* A French author, M. Troilet (Revue Med. September, 1825) has attempted to bring under the notice of the profession, a complaint resembling, but different from, the globus hystericus, so well known as prevailing among nervous females. Cullen mentions this affection, and considers it as hysteria in men. But M. Troilet thinks it absurd to talk of hysteria in the male sex, since the very name shews the disorder to emanate from the uterus. This is ridiculous. We do not see any necessary connexion between the globus hystericus and the uterus in the female, nor do we see any just cause why men should not exhibit the same phenomena. In fact, they do exhibit all the features, occasionally, of the female complaint, modified, of course, by the sex. This, we think, is the case in the present instance; and have little hesitation in coinciding with Cullen, in opposition to M. Troilet. Still the facts are worth attending to, though the theory respecting them may be wrong.

M. Troilet divides the affection into two varieties, according to its seat—the one being in the intestines, the other in the œsophagus.

Those men in whom our author observed the first variety (intestinal) were of advanced age, and the ordinary cause appeared to be daily pressure of some hard body on the abdomen, from peculiar employments. Another cause was unwholesome food and inordinate labour. In the early period of the disorder, the patient only complains of pain in the abdomen and imperfect digestion. After some time the muscular structure of the intestines becomes affected, and the patient begins to feel something like a ball at the bottom and left side of the abdomen, which gradually ascends, in a circuitous direction, towards the stomach, where it discharges itself in gazeous eructations by the mouth, attended with relief. Sometimes these patients vomit up an acid and acrid liquid, accompanied or not by the remains of the food previously taken, when relief also is obtained.

This ball which ascends from below upwards, is generally about the size of one's fist, and is distinguishable by the eye as well as the touch. The patient can arrest its progress, or even force it back a little by firm pressure, which affords him a momentary solace from acute pain. It appears that this ball or globus is evidently formed of air contained in a certain portion of intestine, and which shifts its place in an ascending direction till it reaches the stomach and is discharged. These accessions were generally observed to come on twice a day, with uncertain intervals, and this continued for several days in succession, in one of M. Troilet's patients.

This irritation of the muscular and nervous fibres sometimes rises into phlogosis, and is then attended with constant pain in the intervals of the globus, much increased by pressure, with febrile movement in the pulse &c. Of this form a case will be presently noticed. In other instances, especially where pressure has been the cause of the complaint, and has been of long continuance, scirrhus of the pylorus may be the result. The removal of the causes—the restoration of healthy function in the abdominal viscera—and certain tonic or antispasmodic medicines, are the indications to pursue in such cases. Purgatives were not found advantageous. Aperient lavements were more beneficial.

*Second variety.* This is the globus anti-peristalticus of the œsophagus, which is characterized by the feeling of a ball forming in the epigastrium, and mounting into the chest, where it causes great uneasiness. It ascends to the throat, and there produces a sense of strangulation. In this variety there is no affection, of any kind, of the intestines or abdominal viscera, the disorder appearing to be entirely of the stomach and œsophagus. In the two patients who had this variety, the accessions came on in the evenings, but were excited at any time on taking a full meal, or on being agitated by any strong moral emotion. Our author thinks there is reason to believe that this second variety of globus has been sometimes confounded with angina pectoris. The difference, however, is very great. Not considering this disease as differing essentially from the globus hystericus of females, we shall not enter into the distinctions which our author has attempted to draw between them, but proceed to give a short account of some curious cases of the complaint.

*Case* 1. J. Berrard, 64 years of age, a carpenter, entered the Hôtel Dieu on the 1st September, 1820. He was pale and emaciated. He experienced, in paroxysms, the ascent of a globus from the region of the sigmoid flexure of the colon to the stomach, the ball making several zig-zag turns in its course upwards. When it arrived at the stomach he belched forth much wind, and was then relieved. The tumour was about the size of a cat's head, being tangible and visible. The complaint had existed eight years, and was gradually increasing in severity. He had experienced no illness previously, nor suffered any moral evils. He was obliged to give up work on account of his malady. The paroxysms lasted from one to six or seven hours, during which time he was obliged to sit up, not being able to put himself in the horizontal posture. The burning gas being evolved, a respite of some days ensued, when the same scene again took place. In the course of two months residence in the hospital the paroxysms were rendered much less severe, and at longer intervals, by means of assafœtida taken thrice a day, with warm fomentations to the abdomen, and laxative injections.

*Case* 2. J. Terrabond, aged 42 years, by trade a weaver, had, for several years, been afflicted every evening, after leaving off work, with the globus antiperistalticus already described. These accessions were become more and more distressing. He was often forced to take food, and then to try to dislodge it from his stomach again, for temporary relief. He caught cold in coming from the country to the Hôtel Dieu, and was there seized with a most violent paroxysm, on the 31st October, 1820. He was treated in the hospital with bark, camphor, valerian, and other antispasmodics, by which the disease was mitigated, but not cured. In this state he left the hospital.

*Case* 3. Mossis, a chair-maker, 36 years of age, had suffered strong pressure on the abdomen daily, in the exercise of his trade. Having been affected with a pleurisy, he was cured by the usual means; but afterwards experienced violent accessions of pain in the abdomen, accompanied by the globus antiperistalticus mounting to the stomach, as before-mentioned. In the intervals the pain continued, with some slight febrile symptoms, which gave rise to the fear of inflammation of the intestines. *Leeches, fomentations, abstinence.* The globus took place every day, sensible to the eye and touch, accompanied by pain and tenderness on pressure. Various remedies were now employed of the tonic and antispasmodic kind, with only partial relief. After an increase of the malady, he was again subjected to rigorous diet and antiphlogistic measures, by which the disease was completely removed, and he continued his employment, avoiding, however, to make pressure on the abdomen as formerly. He has since got corpulent. In this case there was, apparently, inflammation added to the other phenomena.

*Case* 4. A. Estrasia, 55 years of age, a pannier-maker, experienced, about five years ago, a sudden pain in the abdomen after lifting a heavy weight. This pain, accompanied by a sense of internal heat, continued for a month, and then disappeared. In the following autumn the pain returned, and lasted the whole winter, attended by much trouble in the digestive function. The disease abated each summer, and again increased in the succeeding two winters. The least error in regimen exasperated the complaint. It was during the last winter only, that the globus antiperistalticus made its appearance. He entered the Hôtel Dieu on the 10th November, 1820. His countenance was pale, and had an expression of suffering—emaciation—abdomen soft, and free from pain in the intervals of the globus—constipation—urine high-coloured and scanty—respiration free—pulse small, slow, and regular. The globus came on every day, but at no particular time. Often there were two or three balls ascending at the same time, with small spaces intervening. When the gas was evolved from the stomach, vomiting often took place. Then there was a respite, and the patient generally went to sleep. The patient could keep no food on his stomach—constant colics came on, and the poor wretch died apparently of inanition on the 30th of November.

*Dissection.* The stomach was of extraordinary dimensions, occupying the epigastrium, both hypochondria, and the umbilical region. It contained air, and a blackish fluid. The parietes were thickened, but pallid. The pyloric orifice was contracted to the size of a goose quill, and its surrounding structure of a fibro-cartilaginous hardness. No trace of inflammation of the mucous membrane. The superior half of the small intestines was contracted, without any augmentation in their parietes. The inferior half was distended with air, and red in some places. The ascending and descending portions of the colon were contracted, red externally, and greyish on the mucous surface. No other alteration of structure in any part of the body.

*Case* 5. This was a man, 51 years of age, who, while lifting a heavy weight, was seized with an acute pain in the loins that lasted, with more or less violence, for three months. It recurred at different times during the succeeding years, particularly in cold weather. The pain shifted occasionally to the hip and right arm. He entered the Hôtel Dieu, on the 8th July, 1820. For fifteen days afterwards the pain was severe, and during that time he was frequently troubled with the sensation of a ball rising from the stomach in the direction of the œsophagus, and causing a feeling of strangulation, when it had risen to the throat. With this affection he had been troubled at times, especially while the pain in the loins was violent, for two years previously. All the functions were natural. The complaint was greatly mitigated by camphor, valerian, and assafœtida—and, perhaps, by an anomalous eruption which came out all over the body after taking some purgative medicine. In this case there was no globus in the intestines, but solely in the œsophagus.

Whether the complaint be viewed as analogous to globus hystericus in women, or, as a peculiar affection resulting from mechanical pressure on the intestines, must be left to the discrimination of the reader. Whatever be its real nature, it has not, as far as our reading extends, been particularly described by any medical author, and, therefore, is deserving of record in this place.

14. *Hæmatoid Tumour of the Brain.* Dr. George Gregory, the able pathologist and physician, has lately published a case of this kind, which shews the necessity of attending to injuries of the head, however trifling may be the attendant symptoms at the time of the accident. A lad, 16 years of age, fell from a ladder in June, 1823, and received a severe blow on the head. He was stunned by the fall, but his medical attendant did not think it necessary to bleed him. Six weeks afterwards he had a strong epileptic fit, after which he was bled and blistered, and so far recovered as to be able to resume his avocations. But he was weak, and affected with head-aches. In May 1825, he had another epileptic seizure of great severity. In June, he took to his bed, and never afterwards left it. Between that time and his death, which happened in October, 1825, he had several paroxysms of epilepsy, and was constantly afflicted with the most excruciating head-aches. An epileptic fit carried him off at last.

On dissection, much bloody serum and some coagula of blood were found in the ventricles of the brain, and in the left anterior lobe was found a cyst " also filled with *coagulated blood* which adhered rather firmly to its internal surface." The parietes of the cavity or cyst were distinct, and could be peeled from the medullary substance of the brain. In the anterior corner of the left ventricle was discovered a hard and fleshy-looking tumour the size of a nutmeg, presenting a laminated structure. It was found to have a small cavity in its centre, and when indurated by immersion in alcohol, it presented appearances " *similar to those of coagulated blood, when treated in a like manner.*"

We submit it to the able pathologist who relates this case, whether both the tumours or cysts were not of the same nature, only varying in age—namely, effusions of blood, similar to what we see in those who have had severe apoplectic seizures? It was a great omission not to mention whether or not this lad ever evinced any hemiplegiac or other paralytic symptom. He certainly had all the requisites for such a phenomenon in the encephalon.

---

15. *Hypertrophia Cerebri.* In a late Number of the *Archives*, M. Scotteten, of Metz, relates a case of this kind, of which we shall record the particulars. A. P. a child of five years of age, presented a great enlargement of the head, which was equal in size to that of an adult. The enlargement had taken place very gradually. For a long time the child had complained of no pain, nor was there any thing particular in the intellectual functions. He was inclined to fall asleep when at rest. All the other functions of the body went on regularly, till the beginning of September, 1823, when he suddenly, and without manifest cause, lost his appetite, became thirsty, complained of pains in the epigastrium, and was more than usually drowsy. This state continued for 15 days, without any alteration. On the 16th day the symptoms became suddenly augmented, and the intellectual functions were entirely abolished. The pupils were dilated, but contracted on the application of a strong light. At mid-day the child died, without convulsion or struggle.

*Dissection.* The skull was from a line to a line and a half in thickness, and this obtained in the places where the fontanelles are usually seen. The dura mater adhered strongly to the skull, but was not altered in structure. The pia mater was much injected with blood, and in some places thickened in its substance. The substance of the brain was very firm. The enlargement of this organ was principally in the posterior part of the hemispheres, and also the superior. It required an incision of three inches in depth to come at the ventricles. There was very little fluid in these cavities. All the other organs of the body were sound except the intestines, which presented some marks of inflammation.

Cases of hypertrophia of the encephalic mass itself, and without effusion in the ventricles, are extremely rare. It is probable that the child, in this instance, did not die of the enlargement of the brain but of the intestinal affection. Whether the inflammation of the membranes of the head or of the intestine was the primary link, it is difficult to say. We think it likely that the meningitis was the primary, and the enteritis the secondary, phlogosis, though the latter may have been the immediate cause of death.

We think it worse than useless in M. Scoutteten to attempt any diagnostic marks of a disease of which there are only two or three on record. We must be contented, for the present, with the facts, but they are too few to support deductions.

---

16. *Encysted Formations.** Three cases of hydatid formations are related by Dr. Bailey, as occurring in the brain, abdomen, and uterus.

*Case* 1. An emaciated female, 26 years of age, came under Dr. B's care in February, 1821. She had pricking pains all over the abdomen, which was tense and enlarged, with partial fluctuation—pulse rapid—respiration quick—secretions regular. The swelling of the abdomen had been on the increase for the last twelve months. Medicine having failed to reduce the swelling, paracentesis was performed, and three gallons of fluid were drawn off. The reduction produced by this evacuation brought into view the figures of several contiguous cysts; but the patient had a temporary relief. In four months she was again tapped in two places, and subsequently, the operation was repeated; but she soon filled, and ultimately died in April, 1822.

On dissection, a great number of cysts was found in the abdomen, containing from a gallon of fluid downwards—besides an immense number of hydatids, varying in size from a pin's head to that of a goose's egg. The cysts contained, some albuminous, and some purulent fluids. Some of them were lined with a layer of coagulable lymph—some with a smooth internal surface—and some with very vascular and even inflamed parietes. Dr. B. observes that this case proves beyond a doubt

---

* Dr F. Bailey. Med. Repos. February, 1826.

the existence of an inflammatory process in the formation of hydatids. The lungs were so jammed up at the superior parts of the thorax as not, at first, to be discoverable.

*Case* 2. Mr. Francis, aged 24 years, became very lethargic about *two months* before the date of report (20th February, 1825) so much so, that he could not sit down ten minutes without falling asleep. This state, unaccompanied by any other morbid symptom, continued two months, when, on lifting a heavy weight, he suddenly experienced a violent pain in the back of his head, followed by strabismus and double vision. He was bled, blistered, and purged, but without relief. Here we are very unaccountably informed that *six months* previously to the date of report, Dr B. was consulted, and found the patient with head-ache, and a painful jarring sensation in the head, on the least motion—pulse quick and bounding—tongue white—strabismus—constipation. Mercury was now tried, and affected the gums, but without benefit. At length the patient became wholly blind, and very deaf—the memory failed, but the other intellectual functions remained unimpaired. He expired on the 10th February, but of what year we are uninformed, the date of report, as beforementioned, standing 20th February, 1825.

*On dissection*, the skull was found very thin, and on the inside scabrous. There was a little serum between the membranes—the substance of the brain was firm, and in the lateral ventricles were found four ounces of clear fluid. On opening the fourth ventricle, a large hydatid was brought into view, (after carefully removing some cerebellic matter) which was three inches in its long diameter, and two in the short. It stretched obliquely across and through the left lobe of the cerebellum to the right. It was found to contain another large cyst, and several very small ones.

*Case* 3. *September* 1, 1824. Mrs. S. aged 46, had been for some months affected with severe pain across the sacrum, extending forwards to the lower part of the abdomen. Latterly these pains were excruciating, and accompanied, occasionally, with uterine hæmorrhage. The constitution became debilitated—the countenance leucophlegmatic—the legs œdematous—respiration difficult—pulse quick and irregular. The dropsical symptoms being removed by diuretics, a violent vomiting came on, probably from the digitalis used. From exposure to cold in this state, she was seized with fever, and the former pains recurred with great violence, and a profuse uterine hæmorrhage. With this discharge a large compact mass of hydatids was passed, resembling a bunch of grapes, and weighing full a pound. In a month the patient was quite well.

Of these cases, that of hydatids in the cerebellum is comparatively rare. The abdominal case is not very uncommon, and hydatids of the uterus are every day seen. Some ingenious physiological and pathological observations are added to the first two cases by Dr. Bailey.

17. *Inflammation of Stomach, Liver, and Duodenum, simulating Yellow Fever.** The following case ought to be read with attention. Joseph Rivot, a soldier, entered the Hospital of Grenoble, on the 12th August, 1825, presenting the following symptoms :—Intense yellow colour of the whole surface of the body—acute pains in the epigastrium and right hypochondrium, as well as in the loins—*frequent vomiting of black matters—numerous stools of the same kind, and extremely fetid*—coldness of the extremities, and generally of the skin—suppression of urine—*tongue white and moist*—thirst urgent—pulse small and contracted—prostration of strength, with stupor and dejection of mind. He had laboured under an intermittent fever, and had only been confined to bed four days before he entered the hospital. He had complained to the surgeon of the regiment of jaundice. During the four days that succeeded his reception in the hospital with the above symptoms, 20 leeches were twice applied to the epigastrium—and an equal number to the anus. The dysenteric stools were so frequent that they were obliged to *plug the rectum in order to apply the leeches.* "On fut obligé de tamponner le rectum pour appliquer ces sangsües."—cold drink, and starch glysters were employed. All the symptoms continued—the pulse became more and more depressed—and the patient expired on the fifth day after entering the hospital.

*Dissection.* The duodenum appeared to be the organ most affected. An eschar, the size of a crown-piece and encircled with black ulcerations, occupied the inferior and posterior part of this gut. The ductus communis choledochus was triple the natural size, produced by a tumefaction, accompanied by redness of its mucous membrane—the gall-bladder was enormously distended with thick, tenacious, black bile—liver gorged with blood—stomach in some places, especially near the pylorus, almost black—the duodenum streaked with brown, livid, and even black spots, and filled with a blackish fluid—the intestines, great and small, were free from disease. The kidneys were red, and the ureters contained bloody urine. The cranial and thoracic viscera presented nothing remarkable.

M. Broussais, in whose Journal this interesting case is published, admits, (seemingly with reluctance, and with much anxiety to account for it,) that the tongue is not always an index of the state of the stomach, "especially in females"—and also that diarrhœa, and even dysentery, may obtain, without any cognizable traces of inflammation in the great intestines or rectum. In the present case, he says, (and probably with some justice) that the inflammation was so concentrated about the liver, stomach and duodenum, as to draw the blood, as it were, from the other organs, and leave them blanched on dissection. Be this as it may, if the case abovementioned had happened in English Harbour instead of the South of France, it would have been considered as one of genuine yellow fever, whether we view it semeiologically or pathologi-

---

* Observations sur une Gastro-hepato-duodenite simulante la Fievre Jaune. Par le Dr. Angelot.—*Ann. de la Med. Physiol. Octobre,* 1825.

cally. As such, it is calculated to afford useful materials for reflection; and to strengthen our confidence in the depletive practice which our army and navy practitioners have followed for many years past in the East and West Indies. True it is—and melancholy is the truth—that no plan will succeed always in yellow fever, such is the intensity of the cause, and the predisposition of the subject. But, by following a good plan, we may do much good and little injury—whereas, by pursuing an erroneous system, we may do much mischief and no good.

---

18. *Mysterious Death.** "His father had long suffered under an indisposition (even before the time his son could remember) which gave him rather frequent pains, than sickness; and gave him cause to be terrified with the expectation of the stone, without being exercised with the present sense of it; but from the time he was 60 years of age, it increased very much, and four or five years before his death, with circumstances scarce heard of before, and the causes whereof are not yet understood by any physician; he was very often, both in the day and the night, forced to make water, seldom in any quantity, because he could not retain it long enough, and in the close of that work, without any sharp pain in those parts, he was still and constantly seized on by so sharp a pain in the left arm, for half a quarter of an hour, or near so much, that the torment made him as pale (whereas he was otherwise of a very sanguine complexion) as if he were dead; and he used to say, 'that he had passed the pangs of death, and he should die in one of those fits;'—as soon as it was over, which was quickly, he was the cheerfullest man living; eat well such things as he could fancy, walked, slept, digested, conversed with such a promptness and vivacity upon all arguments as hath been seldom known in a man of his age; but he had the image of death so constantly before him in those continual torments, that for many years before his death, he always parted with his son, as to see him no more."—"He came to Salisbury on the Friday before Michaelmas day, in the year 1632;—Monday was Michaelmas day, he went, (then, and in the intermediate time seeming to be in the most confirmed health) to the church to a sermon, where he found himself a little pressed as he used to be, and, therefore, thought fit to make what haste he could to his house, and was no sooner come thither into a lower room, than having made water, and the pain in his arm seizing upon him, he fell down dead, without the least motion of any limb. The suddenness of it made it apprehended to be an apoplexy, but there being nothing like convulsions, or the least distortion or alteration in the visage, it is not like to be from that cause; nor could the physicians make any reasonable guess from whence that mortal blow proceeded.

---

* Singular case of Henry Hyde, Esq. related by his son Edward, Earl of Clarendon, Lord High Chancellor of England, and Chancellor of the University of Oxford. (From his life, written by himself, Oxford, MDCCLIX. pages 16, 17, 18.)

He wanted about six weeks of attaining the age of seventy, and was the greatest instance of the felicity of a country life that was seen in that age."

The above case deserves to be recorded, and not less so because it occurred 194 years ago. It possesses a valuable authenticity, since the case was not narrated by an author anxious to support a theory, or eager to confirm an hypothesis, but by an attentive and affectionate son, remarkable for the acuteness of his talents, and distinguished for the accuracy of his judgment. That Mr. Hyde had long suffered under disease of the prostate gland is probable, while it is equally so that this disease was not very serious, whether confined to that organ, or implicating the bladder, for there was neither marked pain nor constitutional indisposition, although considerable irritability. But whence arose the agonizing pain in the arm consequent on the expulsion of the last drops of urine? To this question a satisfactory answer cannot be expected; yet, perhaps, it sprang from nervous sympathy, because it never occurred at any other period, and thus held the relation of a certain effect from a particular cause. Scarcely will it be denied that Mr. Hyde expired in consequence of the intensity of the pain having totally suspended the powers of the heart; since, in previous paroxysms, he was conscious of approaching dissolution, was invariably "as pale as if he were dead," and which state evidently indicated an impeded action in that viscus and its vessels. G. D.

In the above explanation of our learned and esteemed friend G. D. we agree. We have very little doubt that had an attentive POST MORTEM examination been made, a diseased state of the heart or some of its vessels would have been found. We have seen two instances where attacks of angina pectoris were brought on every time the patient had loose motions, or any degree of griping in the bowels.

